# Genome Sequence of *Phytomonas françai*, a Cassava (*Manihot esculenta*) Latex Parasite

**DOI:** 10.1128/genomeA.01266-16

**Published:** 2017-01-12

**Authors:** Claire E. Butler, Eleanor Jaskowska, Steven Kelly

**Affiliations:** a Department of Plant Sciences, University of Oxford, Oxford, United Kingdom; b Kennedy Institute of Rheumatology, University of Oxford, Oxford, United Kingdom

## Abstract

Here, we report the genome sequence of the cassava (*Manihot esculenta*) latex parasite *Phytomonas françai*. *P. françai* infection is linked with the yield-loss disease “chochamento de raizes” (empty roots) in the Unha variety of cassava, a disease characterized by poor root development and chlorosis of the leaves.

## GENOME ANNOUNCEMENT

The trypanosomatids comprise a monophyletic group of kinetoplastid parasites that have evolved to colonize a diverse range of eukaryotic hosts. Most trypanosomatids are transmitted between hosts by biting insects, and several species cause economic and health losses in developing countries (1). Members of a large and diverse subgroup of plant-infecting trypanosomatids known as *Phytomonas* are relatively poorly understood and comparatively little is known of their biology or how they have adapted to life inside plants (2, 3). Furthermore, comparative studies on *Phytomonas* are limited by the lack of availability of genome resources (2), with genome sequences available for only three species (4, 5) at the time of publication of this announcement.

Here, we report the draft genome assembly of *Phytomonas françai* (taxon ID 1902498). The parasite was originally isolated and characterized from the latex ducts of infected cassava plants (*Manihot esculenta* Crantz) in the 1920s (6). The isolate sequenced here was obtained from the latex ducts of an infected cassava (*Manihot esculenta* Crantz var. Unha) plant in the Espirito Santo region of Brazil in 1986 (7). *P. françai* has been linked with the yield-loss disease known as “chochamento de raizes” (empty roots) in the Unha variety of cassava (7, 8). The disease is characterized by poor root development and chlorosis of the leaves (7, 8). However, the Unha cultivar of cassava that exhibited disease when infected with *P. françai* is no longer widely farmed in Brazil, and there have been no reports of empty roots since 1980 (3). Thus, although *P. françai* has been repeatedly isolated from cassava, it appears to pose little risk to crop yield.

Whole-genome sequencing was performed on an Illumina HiSeq using a five-library approach. This comprised three libraries of 100-bp paired-end reads (insert sizes of 170 bp, 500 bp, and 800 bp) and two libraries of 100-bp mate-pair reads (insert sizes of 2,000 bp and 5,000 bp). Quality-filtered reads were assembled using ALLPATHS-LG (9) into scaffolds. Gene models were predicted using AUGUSTUS (10) and clustered into orthogroups using OrthoFinder (11). The final draft assembly contained 7,108 putative protein-coding genes distributed on 96 scaffolds with a scaffold *N*_50_ of 784 kb and a total assembly length of 17.7 Mb. RNAseq was also performed to enable abundance estimation of genes and identification of mRNA processing sites (12). This resource is provided to expand the molecular sequence data available for comparative studies in this globally important and poorly understood group of parasites.

### Accession number(s).

This whole-genome shotgun project has been deposited in GenBank under the accession number MJCC00000000. Raw genome reads have been deposited in NCBI SRA under the accession numbers SRR4244756 to SRR4244760. RNAseq reads are available through EBI under the accession number E-MTAB-5110.
